# *Mycobacterium microti* Infection in Dairy Goats, France

**DOI:** 10.3201/eid2203.151870

**Published:** 2016-03

**Authors:** Lorraine Michelet, Krystel de Cruz, Yohann Phalente, Claudine Karoui, Sylvie Hénault, Marina Beral, María L. Boschiroli

**Affiliations:** Agence Nationale de Sécurité Sanitaire de l’Alimentation, de l’Environnement et du Travail, Maisons-Alfort, France (L. Michelet, K. de Cruz, Y. Phalente, C. Karoui, S. Hénault, M.L. Boschiroli);; Direction Régionale de l'Agriculture et de la Forêt, Bourgogne, Dijon, France (M. Beral)

**Keywords:** Mycobacterium microti, bacteria, tuberculosis and other mycobacteria, infection, dairy goats, bovine tuberculosis, France

**To the Editor:** *Mycobacterium microti* is a member of the *Mycobacterium tuberculosis* complex (MTBC). This complex also includes *M. tuberculosis*, which causes human tuberculosis, and *M. bovis* and *M. caprae*, which cause bovine tuberculosis. *M. microti* was initially described as a pathogen of small rodents and also frequently affects domestic animals, especially cats, and has also been described in wildlife, especially wild boars and badgers (*1*). This mycobacterium has also been involved in human pulmonary tuberculosis cases, which highlights its potential zoonotic risk (*2*). We report a case of *M. microti* infection in a dairy goat herd, which underlines the risk for confounding bovine tuberculosis diagnosis and potential consequences for livestock management.

France has been considered officially free of bovine tuberculosis by the European Union since 2001. Surveillance of this disease is based on antemortem testing with tuberculin skin tests and on systematic postmortem inspection at abattoirs through sanitary inspection of carcasses to detect bovine tuberculosis–like lesions. However, because bovine tuberculosis evolves in an insidious manner and antemortem or postmortem diagnostic tests are not efficient for detecting latently infected animals, an infected herd may remain unidentified for long periods and can be responsible for contamination of other herds by animal movement or contact with animals of neighboring herds. For this reason, to detect other potentially associated cases, investigations in herds epidemiologically linked to the index outbreak are also performed, either through skin testing or through diagnostic culling of those animals introduced from the infected herd or any other animal with a skin test–positive result.

This case of bovine tuberculosis in a goat was reported in a region of the Alps Mountains in France. The herd was composed of 140 dairy goats, which were used for raw milk cheese production. Goats were semi-extensively bred and kept in pastures *>*6 months per year. Investigations conducted after identification of a case of bovine tuberculosis in a neighboring cattle herd infected with *M. bovis*, in which the index case was identified at an abattoir, highlighted the epidemiologic link with the goat herd because animals in both populations shared the same pastures. Thus, the goat herd was subjected to single intradermal tuberculin tests.

Three adult goats (goats A, B, and C) showed positive results and were culled for direct diagnosis. Goats A and B showed no lesion at abattoir inspections, but goat C had bovine tuberculosis–like lesions on the retropharyngeal and mediastinal lymph nodes. Retropharyngeal, tracheobronchial, and mediastinal lymph nodes were sampled from the 3 goats. Retromammary lymph nodes were also sampled from goats B and C (both females). Lesions from goat C were examined by histopathologic analysis and showed a profile suggestive of bovine tuberculosis with necrosis, Langhans giant cells, and few acid–alcohol-resistant bacilli by Ziehl-Neelsen staining (Figure). All samples were subjected to bacterial culture and molecular diagnosis (*3*).

**Figure:**
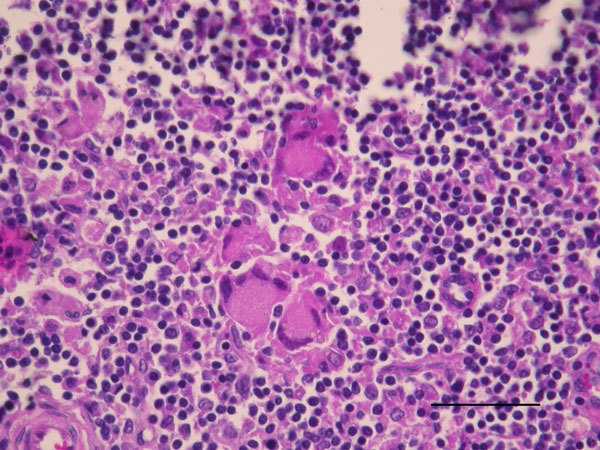
Goat lymph node granuloma with numerous Langhans-type multinucleated giant cells from a goat in France infected with *Mycobacterium microti* (hematoxylin and eosin stain). Scale bar indicates 50 μm.

Although after 3 months culture results were negative for all samples, DNA extracted from the retropharyngeal lymph node of goat C showed a positive PCR result for MTBC DNA by the LSI VetMAX *Mycobacterium tuberculosis* Complex Real-Time PCR Kit (Thermo Fisher Scientific, Villebon sur Yvette, France). Further characterization of this DNA was performed by using molecular analysis specific for the regions of difference, which enables differentiation of MTBC members (*4*), and spoligotyping (*5*).

The infectious agent from goat C was identified as *M. microti* spoligotype SB0118. Moreover, a bovine tuberculosis investigation in wildlife (*6*) identified *M. microti* spoligotype SB0118 infection case in a dead badger found 8 km from the goat farm. Thus, the long time during which goats remain in pastures might have favored environmental contamination by interaction with wildlife. Furthermore, an additional case of *M. microti* infection in a cat reported in 2011 in the same region also had the SB0118 spoligotype (*7*), which demonstrated that this bacillus is actively circulating in animals from this area.

*M. microti* was previously isolated on the basis of a skin test–positive result for cattle in the United Kingdom (*8*), which demonstrated the risk for infection in livestock. These findings raise concern on reliability of diagnostic tests used for bovine tuberculosis surveillance. *M. microti*, which is phylogenetically similar to *M. bovis* or *M. caprae* and widely disseminated in the environment, could be responsible for misleading diagnostic results, as demonstrated in this study.

Highly specific tests are needed to accurately identify *M. bovis* (or *M. caprae*) infection at antemortem examination through use of specific antigens, such as ESAT 6 and CFP10, which are absent in *M. microti* and are currently used in the interferon-γ test in France (*9*). In addition, at postmortem diagnosis, use of specific molecular tools capable of rapidly distinguishing members of the MTBC should be considered. Histopathologic analysis lacks specificity, and obtaining results for bacterial culture takes too much time for these particularly slow-growing and fastidious mycobacteria.

*M. microti* has already been reported to cause tuberculosis in immunocompromised and immunocompetent patients in France (*10*). Thus, potential risk for infection of humans by consumption of raw goat milk cheese cannot be ruled out.
